# How well do elderly patients do after total knee arthroplasty in the era of fast-track surgery?

**DOI:** 10.1186/s42836-020-00037-5

**Published:** 2020-06-22

**Authors:** Amy Cheung, Henry Fu, Man Hong Cheung, Wai Kwan Vincent Chan, Ping Keung Chan, Chun Hoi Yan, Kwong Yuen Chiu

**Affiliations:** 1 Department of Orthopaedics and Traumatology, Queen Mary Hospital, Hong Kong, China; 2 Department of Orthopaedics and Traumatology, University of Hong Kong, Hong Kong, China

**Keywords:** Total knee arthroplasty, Elderly, Old age, Enhanced recovery after surgery (ERAS), Fast track arthroplasty

## Abstract

**Introduction:**

Total knee arthroplasty (TKA) in the elderly population is becoming increasingly prevalent. This study aimed to compare outcomes of patients aged ≥80 years with those aged < 80 years at time of TKA and to assess the effect of fast track peri-operative care on outcomes in the elderly.

**Materials and methods:**

422 TKAs were performed in aged ≥80 at the time of surgery between 2009 and 2018. A control group aged < 80 years (37–79 +/− 7.6) was established. Peri-operative mortality, complications, 30-day re-admission, length of stay (LOS) and rehabilitation parameters were recorded.

**Results:**

Mean age at operation for the ≥80’s group and control group was 82.7 (80–93+/− 2.5) and 69.3 (37–79+/− 7.6) years respectively. Post-operative Knee Society Functional Assessment (KSFA) scores were higher in the control group (49 *vs*. 57, *p* = 0.003). Average LOS was longer in the ≥80’s group (17.2 *vs*. 12.4 days respectively, *p* < 0.01). Mortality within 3 months of operation was 0.7% in the ≥80 group and 0% in the control group. Incidence of complications was comparable between the two groups at 12.8 and 12.9% for the group ≥80’s and control groups respectively (*p* = 0.962). Patients ≥80 years, receiving fast track peri-operative care had significantly shorter LOS and higher post-operative KSFA scores at all time points post-operation and shorter LOS (*p* < 0.01) compared to those who received conventional rehabilitation. LOS was longer in the ≥80’s group, which was likely related to higher levels of comorbidities. Complications were comparable in the two groups but were more severe in the elderly. Mortality rate after TKA was very low even in those over the age of 80. Younger patients benefited more in terms of functional improvement after TKA.

**Conclusion:**

TKA is a safe and efficacious procedure for the elderly. More severe complications, longer length of stay and smaller gains in functional improvement can be expected in the elderly compared to younger patients. Fast track peri-operative care is useful in improving outcomes after TKA for elderly patients.

## Introduction

By 2041, 30.2% of Hong Kong’s population will be aged 65 years or older. With similar trends of population aging worldwide, the prevalence of total knee arthroplasty (TKA) being performed in elderly patients can be expected to increase. In Hong Kong, from 2000 to 2009, the number of patients aged over 80 years undergoing TKA have been on the rise [1]. Traditionally, TKA in octogenarians is associated with higher mortality and complication rates, longer rehabilitation periods and longer length of hospital stay [2]. Currently, fast track arthroplasty and the adoption of the principles of enhanced recovery after surgery (ERAS) have led to improvement in rehabilitation outcomes after TKA with shorter length of hospital stay and hastened recovery [3, 4]. Fast track arthroplasty involves the use of a multi-disciplinary approach with the aim of reducing the length of hospital stay, morbidity, and convalescence, without an increase in readmission rates or safety issues [5]. Intra-operative measures to effect this include the use of spinal anaesthesia, local analgesic infiltration, no drains and compression bandaging. Post-operative measures include early initiation of thromboembolic prophylaxis, multi-modal opioid sparing analgesic regimens and accelerated rehabilitation and discharge. Fast track rehabilitation protocols involve the use of early mobilization and concerted efforts to allow for early rehabilitation with the aim to decrease recumbency-related complications such a thromboembolic events, urinary tract and chest infections [6]. However, it is currently still unclear whether elderly patients can benefit from fast track regimens and there are few studies in the literature examining this specifically.

The aim of this study was to examine and compare the results after TKA in patients ≥80 years and patients < 80 years at time of TKA. Furthermore, this study also sought to determine whether there are any significant differences in rehabilitative outcomes in elderly patients who undergo rehabilitation using a fast track regimen compared to patients undergoing rehabilitation using a standard regimen.

## Methods

Between 2009 and 2018, a total of 422 TKA were performed in 321 patients aged ≥80 years (elderly group) at the time of operation. A control group was set consisting of patients < 80 years at the time of operation who were operated on in the same period of time as their elderly counterparts. Baseline pre-operative parameters, including the Charlson Comorbidity Indices (CCI) [7], Knee Society Knee Score (KSKS) and Knee Society Knee Functional Assessment (KSFA) scores were recorded. Post-operative outcome measures, including presence of peri-operative mortality and complications and 30-day re-admission, were recorded. Complications were graded according to the Clavien-Dindo grading system [8] for surgical complications.

Rehabilitation parameters, including KSKS and KSFA scores, were recorded at 6 weeks, 3 months and at latest follow-up.

Age, CCI, KSKS and KSFA scores were all found to be non-normally distributed. Mann-Whitney U and Chi-square tests were therefore used for comparison between groups.

## Results

Average age at the time of operation for the elderly group and control groups were 82.7 and 69.3 years respectively. Table 1 outlines the baseline characteristics of the elderly and control groups. Baseline KSFA and CCI of the controls were statistically significantly more favorable as compared to the elderly group. Baseline KSKS, on the other hand, did not differ significantly between the two groups.

**Table 1:** The baseline characteristics of the elderly and control groups

	≥80 years (*n* = 423)	< 80 years (*n* = 215)	Significance
Average age	82.7 (80–93 +/− 2.5)	69.3 (37–79 +/− 7.6)	*p* < 0.01
Male: female	1:3	1:3.1	
Pre-operative KSKS	45.4	42.3	*p* = 0.085
Pre-operative KSFA	39.7	47.8	*p* < 0.01
Charlson Comorbidity Index	4.8	3.3	*p* < 0.01

**Fig. 1:**
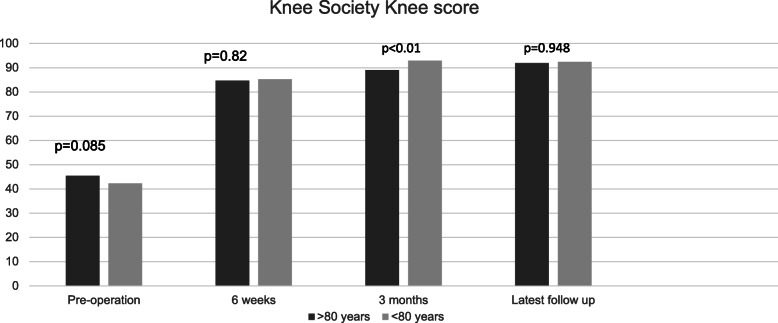
KSKS at various time points for both the elderly and control groups

**Fig. 2:**
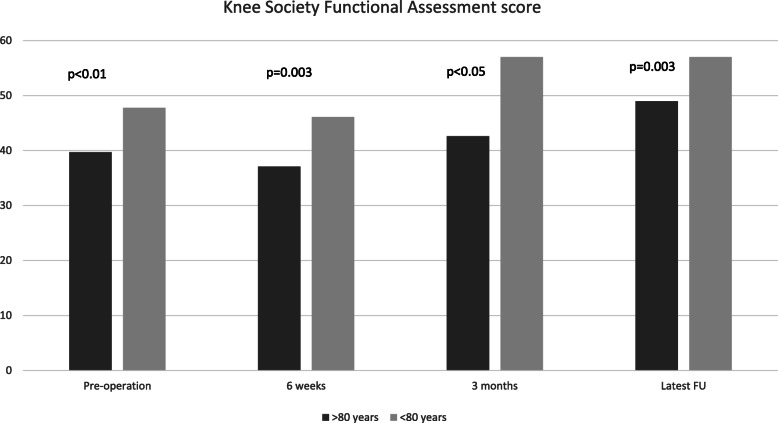
KSFA at various time points for both elderly and control groups

Post-operative KSKS scores were significantly higher at 3 months and at latest follow-up for the control group compared to the elderly group (Fig. 1). Similarly, at all post-operative time points, KSFA scores were significantly higher in the control than in the elderly group (Fig. 2). Length of hospital stay was significantly longer in the elderly group than in the control group (17.2 days *vs*. 12.4 days, *p* < 0.01).

Incidence of complications was similar between the elderly and control groups (12.8% *vs*. 12.9%, *p* = 0.962). However, when the severity of complications was taken into account, those in the elderly group had more severe complications than those in the control group (sum of ranks 1769 *vs*. 1157, U = 338, *p* = 0.01). Table 2 details the incidence of each type of complication in both the elderly and control groups.

**Table 2:** The incidence of different types of complications in the elderly and control groups

Type of complication	Elderly patients (incidence, %)	Control group (incidence, %)
Cerebrovascular accidents	0.2	0
Cardiovascular events	1.8	0.5
Delirium	0.7	0.5
Deep vein thrombosis	0.2	0
Fracture	0.7	2.5
Intestinal obstruction	0.7	0
Peri-prosthetic Joint Infection (PJI)	1.1	3
Non-PJI sepsis	0.4	0
Nerve palsy	0.2	0
Medial collateral ligament injury	0.2	0.5
Peptic ulcer	0.4	0
Extensor mechanism injury	0	0.5

Mortality rates within 30 days of operation were 0.7 and 0% in the elderly and control groups respectively.

**Fig. 3:**
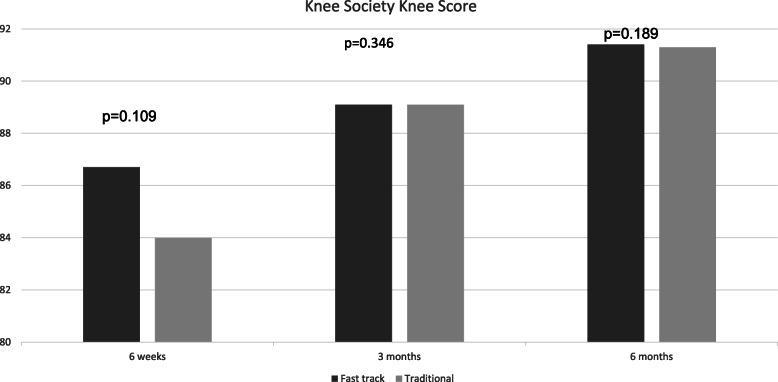
KSKS of fast track group compared to traditional rehabilitation group at various time points

**Fig. 4:**
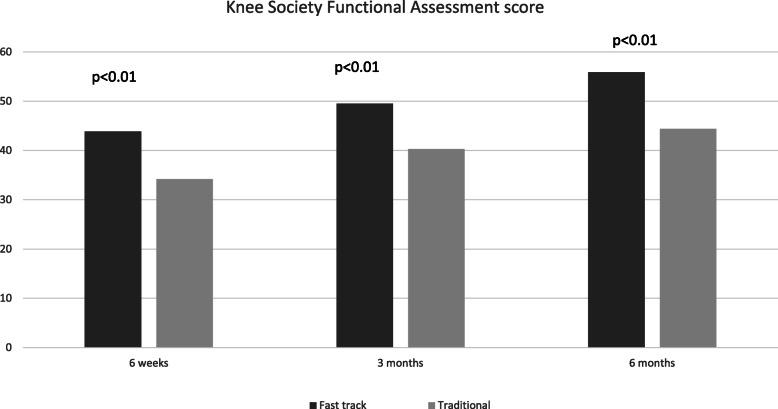
KSFA scores of the fast track group compared to traditional rehabilitation group at various time points

Table 3 details the baseline characteristics of elderly patients undergoing traditional rehabilitation and those receiving a fast-track regimen. Elderly patients undergoing rehabilitation via a fast-track regimen had statistically significantly shorter length of hospital stay than their elderly counterparts undergoing a traditional rehabilitation regimen (12 days *vs*. 19.4 days, *p* < 0.01). Although KSKS did not differ significantly between the two groups at all time points after operation (Fig. 3), KSFA scores were significantly more favorable in patients on the fast-track rehabilitation than in those receiving traditional rehabilitation at all time points post-operation (Fig. 4). Furthermore, no significant difference was present in the incidence of complications between the fast track rehabilitation group and the traditional rehabilitation group (13.4% *vs*. 12.5%, *p* = 0.834).

**Table 3:** The baseline characteristics of the elderly patients in both the traditional rehabilitation and fast track rehabilitation groups

	Traditional rehabilitation group (*n* = 296)	Fast track rehabilitation group (*n* = 126)	Significance
Average age	82.3 (80–93 +/− 2.3)	83.8 (37–79 +/− 2.7)	*p* = 0.000
Male: female	1:3	1:3.1	
Pre-operative KSKS	43.7	49.6	*p* = 0.002
Pre-operative KSFA	38.7	42.1	*p* = 0.004
Charlson Comorbidity Index	4.8	4.9	*p* = 0.202

## Discussion

Overall, mortality rates were very low after TKA in both the elderly and control groups, at 0 and 0.7% respectively. This remarkably low mortality rate found in this study could be attributed to stringent pre-operative assessment and optimization of medical comorbidities as well as careful peri-operative management. This overall low incidence of mortality was also found in similar studies [9, 10]. However, significantly increased mortality has also been found in elderly patients [10, 11].

The overall complication rate of both groups was similar to that found in the study by Pavone *et al* [12]. Although complication rates were similar in both the elderly and control groups, those in the elderly group tended to have more severe complications. This is consistent with the findings of Easterling *et al*, who found that the risk of complications after TKR increased markedly in patients aged over 80 years [13].

However, this finding contrasts with that found in the study of Kennedy *et al*, where, interestingly, more severe complications were found in younger patients compared to elderly patients [14]. The authors attributed this finding to the more thorough use of pre-emptive measures against infection and thrombotic events in high-risk surgical candidates. All patients in this study underwent a standardized protocol for pre-emptive measures against both infective and thromboembolic complications. Baseline CCI was significantly higher in the elderly group compared to the control groups. Therefore, this difference in the severity of complications observed in the elderly and control group may perhaps reflect the underlying differences in medical comorbidities between the two groups.

Length of hospital stay was significantly longer in the elderly group of patients than in the control group. This finding is in agreement with that found in similar studies examining this outcome measure [2, 15].

KSKS was not found to be significantly different between elderly and younger patients post-operation. However, KSFA was significantly higher in the control group at all time points after operation. Therefore, although pain relief and objective physical assessment of the two groups did not differ post-operation, the resultant function was significantly different. This might reflect the inherent differences between these two groups of patients, owing to higher levels of comorbidities in the elderly, and highlight the additional challenges involved in rehabilitating elderly patients [16]. This finding was in keeping with that of other studies examining this [14, 17].

In this study, elderly patients undergoing a fast track regimen had superior functional outcomes at all time points after operation and shorter length of hospital stay with similar complication rates. This highlights the effectiveness of the adopting principles of ERAS in improving patient outcomes in modern age arthroplasty. The results of this study concurs with other studies which have demonstrated the superiority of fast track regimens over traditional regimens in elderly patients [18].

There is currently paucity of literature looking specifically into the effect of adopting fast-track rehabilitative concepts in the elderly. Further high-level research should be directed at determining the effectiveness of such strategies in the elderly population.

A major weakness of this study is that it was of retrospective design. Therefore, it was not possible to control for various confounding variables which may have led to the observed differences between the elderly and control groups, such as underlying comorbidities. However, this weakness may also serve to gain insight into the inherent differences in characteristics of the elderly compared to younger patients, which may be beneficial in aiding risk stratification and decision-making before surgery.

This study adds to the sparse body of literature examining the effect of adopting the principles of ERAS in TKA in elderly patients.

## Conclusion

In conclusion, TKA is a safe and efficacious procedure even in patients aged ≥80 years. However, longer length of hospital stay with smaller gains in functional improvement as well as more severe complications can be expected in this population. Furthermore, fast track peri-operative care is safe and beneficial in shortening the length of hospital stay and improving function in elderly patients.

## Data Availability

The datasets used and/or analyzed during the current study are available from the corresponding author on reasonable request.

## Abbreviations

TKA: Total knee arthroplasty
ERAS: Enhanced recovery after surgery
CCI: Charlson Comorbidity Index
KSKS: Knee Society Knee Score
KSFA: Knee Society Functional Assessment

## References

[1] Yan CH, Chiu KY, Ng FY (2011). Total knee arthroplasty for primary knee osteoarthritis: changing pattern over the past 10 years. Hong Kong Med J.

[2] Clement ND, MacDonald D, Howie CR, Biant LC (2011). The outcome of primary total hip and knee arthroplasty in patients aged 80 years or more. J Bone Joint Surg (Br).

[3] Khan SK, Malviya A, Muller SD, Carluke I, Partington PF, Emmerson KP (2014). Reduced short-term complications and mortality following enhanced recovery primary hip and knee arthroplasty: results from 6,000 consecutive procedures. Acta Orthop.

[4] Stambough JB, Nunley RM, Curry MC, Steger-May K, Clohisy JC (2015). Rapid recovery protocols for primary total hip arthroplasty can safely reduce length of stay without increasing readmissions. J Arthroplast.

[5] Kehlet H, Dahl JB (2003). Anaesthesia, surgery, and challenges in postoperative recovery. Lancet..

[6] Larsen K, Hansen TB, Thomsen PB, Christiansen T, Soballe K (2009). Cost-effectiveness of accelerated perioperative care and rehabilitation after total hip and knee arthroplasty. J Bone Joint Surg Am.

[7] Charlson ME, Pompei P, Ales KL, MacKenzie CR (1987). A new method of classifying prognostic comorbidity in longitudinal studies: development and validation. J Chronic Dis.

[8] Dindo D, Demartines N, Clavien PA (2004). Classification of surgical complications: a new proposal with evaluation in a cohort of 6336 patients and results of a survey. Ann Surg.

[9] Parvizi J, Sullivan TA, Trousdale RT, Lewallen DG (2001). Thirty-day mortality after total knee arthroplasty. J Bone Joint Surg Am.

[10] Chikuda H, Yasunaga H, Horiguchi H, Takeshita K, Sugita S, Taketomi S (2013). Impact of age and comorbidity burden on mortality and major complications in older adults undergoing orthopaedic surgery: an analysis using the Japanese diagnosis procedure combination database. BMC Musculoskelet Disord.

[11] Kuperman EF, Schweizer M, Joy P, Gu X, Fang MM (2016). The effects of advanced age on primary total knee arthroplasty: a meta-analysis and systematic review. BMC Geriatr.

[12] Pavone V, Boettner F, Fickert S, Sculco TP (2001). Total condylar knee arthroplasty: a long-term followup. Clin Orthop Relat Res.

[13] Easterlin MC, Chang DG, Talamini M, Chang DC (2013). Older age increases short-term surgical complications after primary knee arthroplasty. Clin Orthop Relat Res.

[14] Kennedy JW, Johnston L, Cochrane L, Boscainos PJ (2013). Total knee arthroplasty in the elderly: does age affect pain, function or complications?. Clin Orthop Relat Res.

[15] Maempel JF, Riddoch F, Calleja N, Brenkel IJ (2015). Longer hospital stay, more complications, and increased mortality but substantially improved function after knee replacement in older patients. Acta Orthop.

[16] Kennedy LG, Newman JH, Ackroyd CE, Dieppe PA (2003). When should we do knee replacements?. Knee.

[17] Williams DP, Price AJ, Beard DJ, Hadfield SG, Arden NK, Murray DW (2013). The effects of age on patient-reported outcome measures in total knee replacements. Bone Joint J.

[18] Pitter FT, Jorgensen CC, Lindberg-Larsen M, Kehlet H (2016). Postoperative morbidity and discharge destinations after fast-track hip and knee arthroplasty in patients older than 85 years. Anesth Analg.

